# Deoxycholic Acid as a Minimally Invasive Treatment for a Giant Dorsal Lipoma: A Case Report

**DOI:** 10.7759/cureus.90793

**Published:** 2025-08-23

**Authors:** Connor Latterman, Joyce I Imahiyerobo

**Affiliations:** 1 Dermatology, Vibrant Dermatology, Dedham, USA

**Keywords:** back lipoma, deoxycholic acid, giant lipoma, lipoma, minimally invasive therapy

## Abstract

A 39-year-old woman presented with a 14.0cm by 10.0cm lipoma on the left posterior-lateral flank. Conventional treatment for lipomas includes excision, but this poses a risk of extensive scarring in patients with large lipomas. Deoxycholic acid (DCA) injections are a viable non-surgical alternative to reduce the size of lipomas. The patient received two rounds of intralesional DCA injections three months apart, with each using 8.5mL of 10mg/mL solution. Four months after beginning treatment, she noticed a 50% reduction in the size of the lesion. This case highlights the potential for using DCA injections as a safe, effective treatment to significantly reduce the volume of large lipomas without surgical scarring.

## Introduction

Lipomas are subcutaneous tumors composed of adipose cells, most commonly found in the head, neck, shoulders, and back of patients [1]. While they can occur at any age, they most often first appear in patients between 40 and 60 years of age and grow slowly over time. Usually, they present as asymptomatic, round, mobile masses with a soft, doughy feeling, but they can rarely be associated with conditions such as multiple hereditary lipomatosis, Gardner syndrome, adiposis dolorosa, and Madelung disease [2]. Lipomas are most often left alone unless they become painful or start growing rapidly. Conventional treatment methods include direct excision, liposuction, laser extirpation, or deoxycholic acid (DCA) injections [3]. Small lipomas (less than 4 centimeters in size) can be surgically excised, leaving a small scar; however, excising lipomas of greater size poses a risk of extensive scarring. This case report aims to illustrate the successful use of DCA injections to significantly reduce the size of a large lipoma, preventing the need for surgical excision and associated scarring. It highlights a minimally invasive alternative for managing cosmetically distressing large lipomas.

## Case presentation

**Figure 1:**
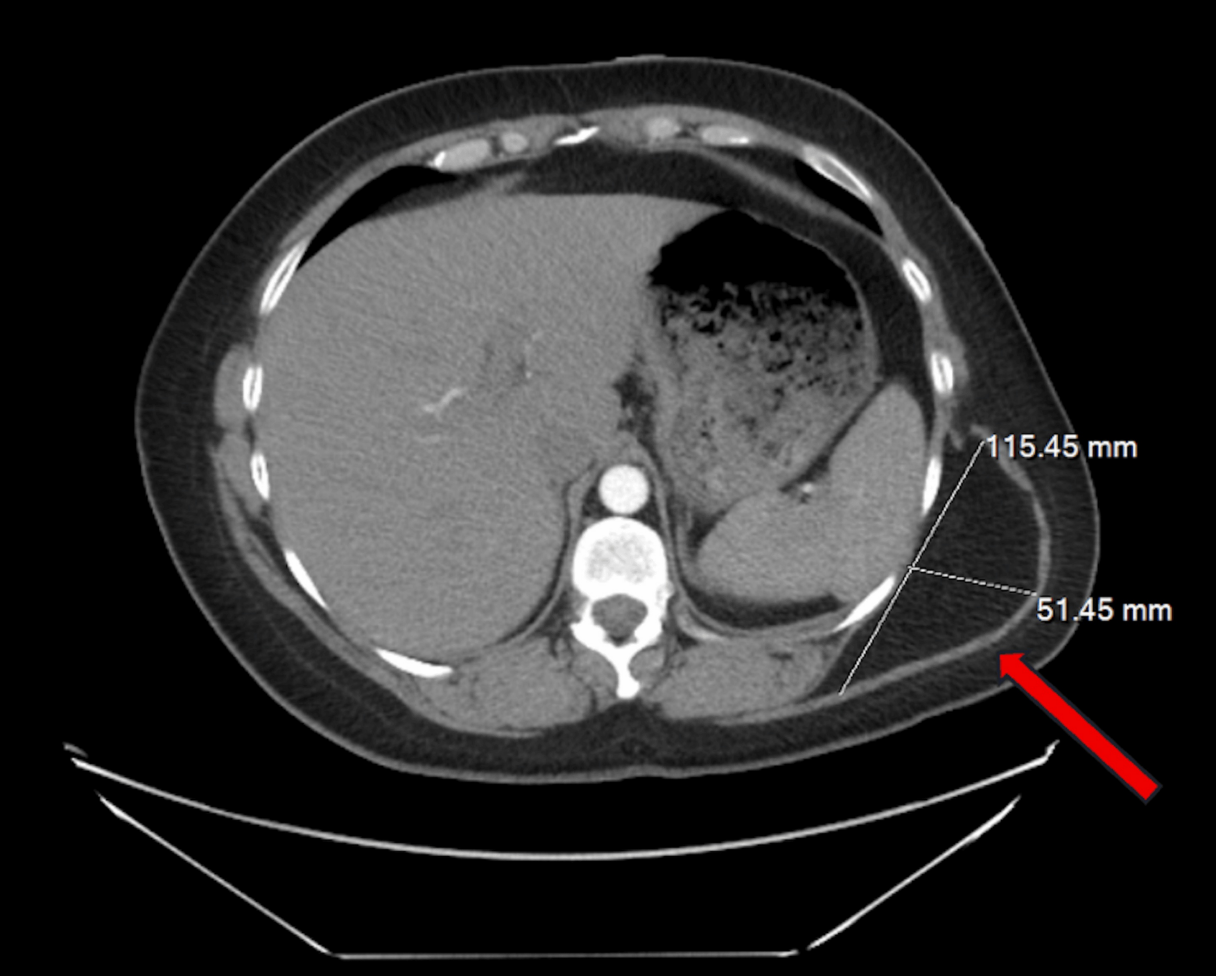
CT image of an incidental finding of a large lipoma measuring 11.5cm by 5.1cm, along the left posterior lateral flank region, deep to the latissimus dorsi muscle (arrow).

**Figure 2:**
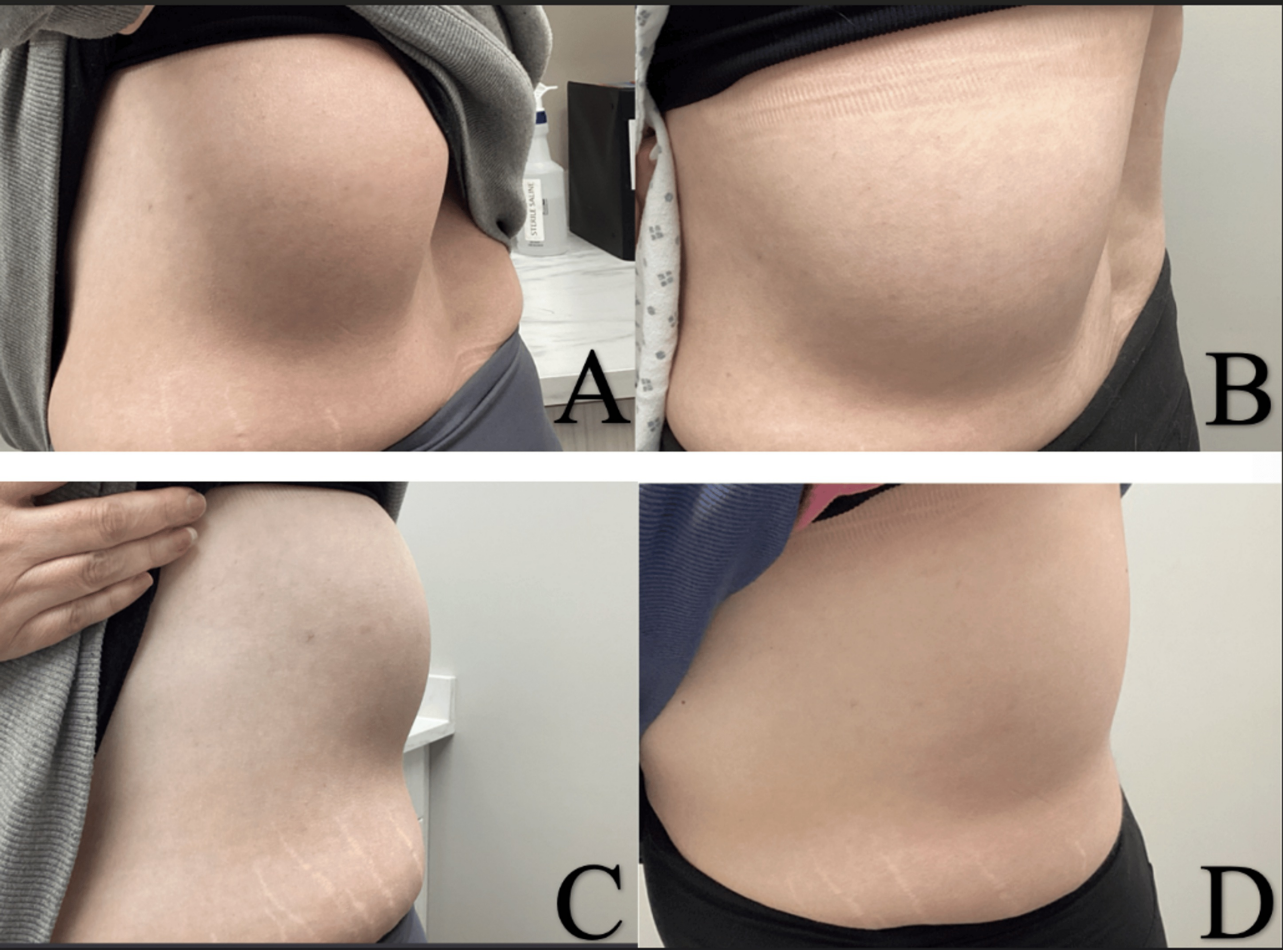
Baseline pretreatment evaluation of the lesion. (A and C) A 14cm by 10cm lipoma on the left posterior lateral flank. (B and D) Follow-up evaluation after two deoxycholic acid treatments three months apart with a significant reduction in the size of the lesion.

A 39-year-old woman presented to the clinic with a painless, slow-growing tumor on her left posterior lateral flank. Initial evaluation revealed a 14.0cm by 10.0cm soft, nontender, mobile subcutaneous mass (Figure 1). Five years prior, in 2020, a computed tomography (CT) angiogram was conducted with an incidental finding of a large lipoma measuring 11.5cm by 5.1cm along the left posterior lateral flank region deep to the latissimus dorsi muscle (Figure 2). The patient requested that the lesion be excised due to its size and cosmetic distress. She was extensively counseled on the risk of scarring if it were to be excised. Instead, the patient opted for a series of DCA injections as a minimally invasive treatment to significantly decrease the size of the lipoma in the office.

The patient received two treatments three months apart. After obtaining informed consent, the first procedure was done at her initial visit. A skin marking grid template was applied to the skin, and 8.5mL of DCA (10mg/mL) was injected with 0.2mL per injection site using 1mL syringes with a 30-gauge needle. After the injection, the lipoma was massaged. Our patient tolerated the treatment well and experienced mild, sustained swelling for one week. The procedure was repeated three months later. After the second procedure and four months since beginning treatment, the patient noticed a 50% reduction in the size of the lesion. The patient reported satisfaction with the results and thus, further treatment with surgical excision was deferred.

## Discussion

Lipomas can be found in any organ, but they are most commonly located subcutaneously on the neck and trunk, rarely involving the deep muscular plane [4]. Non-surgical lipoma treatments exist; however, they are more effective in smaller lipomas not extending to the deep muscular plane, like our patient. Steroid injections can result in the shrinking of small lipomas by causing local fat atrophy, but they rarely eliminate the lipoma [5]. Additionally, liposuction can be used to remove small lipomas, but complete elimination is difficult, especially in larger lipomas. Due to the large size and depth of the latissimus dorsi muscle, treatment options included excision with the downside of extensive scarring or DCA injections to dramatically shrink the size of the lipoma.

DCA is a synthetic version of the endogenous bile acid naturally found in the liver [6]. Bile acids are steroid molecules synthesized from cholesterol and transported to the small intestine to emulsify aggregates and transport lipids and fast soluble vitamins [7]. DCA has fat emulsification properties most commonly used for non-surgical submental fat reduction, improving mandibular definition [8,9]. Injection causes cell membrane disruption (oncosis), acute inflammation, and fat necrosis in adipocyte cells one to three days post-treatment [10,11]. When injected into subcutaneous fat, protein-rich tissues like muscles, blood, and skin are unaffected [6]. One week later, lipid macrophages are recruited to the area, causing long-lasting fat reduction after fat is released into the extracellular matrix and metabolized by the liver [6].

Due to the similar cellular composition between fat deposits and lipomas, DCA has been experimentally used to treat small lipomas. Unlike surgical excision, which carries the risk of infection and significant scarring, especially with large lipomas, DCA injections offer a less invasive alternative with minimal downtime. While DCA is FDA-approved for the reduction of submental fat, its off-label use in lipomas has been detailed in several case reports and small studies.

A study by Rotunda et al. demonstrated a mean reduction in size of 75% of 12 small lipomas after one to four DCA treatments [12]. One case report details the use of DCA for a large lipoma of 9.5cm x 8.0cm, but there are no reported successful cases of using DCA for a lipoma as large as our patient’s. This case adds to the literature demonstrating its effectiveness in a much larger lesion (14cm x 10cm). Further reports or studies should prioritize extended follow-ups to assess recurrence rates, sustained cosmetic improvement, and any delayed adverse effects.

## Conclusions

This case details the use of DCA as a minimally invasive technique to significantly reduce the size of a large lipoma (14.0cm x 10.0cm). Although the results were slower and less dramatic than excision, the patient experienced significant cosmetic improvement and increased self-confidence. This report adds to the growing evidence of using DCA to treat lipomas in a minimally invasive and cosmetically appealing manner. However, further research is necessary to determine long-term treatment outcomes for large lipomas and possible adverse reactions such as site burning, pain, or erythema.
